# Effects of Concurrent Strength and HIIT-Based Endurance Training on Physical Fitness in Trained Team Sports Players: A Systematic Review and Meta-Analysis

**DOI:** 10.3390/ijerph192214800

**Published:** 2022-11-10

**Authors:** Jian Kang, Zhijing Ye, Xinxing Yin, Changjing Zhou, Bo Gong

**Affiliations:** 1 School of Elite Sport, Shanghai University of Sport, Shanghai 200438, China; 2 Shanghai Shenhua FC, No. 2600 Hu Nan Road, Pudong District, Shanghai 201315, China; 3 School of Marxism Studies, Xi’an Jiaotong University, Xi’an 710049, China

**Keywords:** concurrent training, strength training, endurance training, physical fitness, interference

## Abstract

Background: Concurrent strength and HIIT-based endurance training (CT) has merit in time-saving in team sports. However, the effect of CT on physical fitness remained equivocal. This meta-analysis aimed to determine whether CT would produce an interference effect on the development of physical fitness when compared to strength training (ST) or HIIT-based endurance training (HET) alone in trained team sports players. Methods: A total of 2478 studies from three databases were screened. 52 full texts were reviewed. Seven studies were finally included and then subgroups were used for quantitative analysis. Results: Compared to ST alone, CT had a significant effect on the development of maximal lower-body strength in trained team sports players (MD 4.20 kg, 95% CI 0.71–7.68, *p* = 0.02, I^2^ = 20%), but there was no significant difference between the groups on training adaptation in lower-body power (SMD 0.08, 95% CI −0.23–0.39, *p* = 0.62, I^2^ = 26%). Furthermore, a sub-group analysis based on the internal organization order of CT revealed that there was no statistically significant subgroup effect between CT and ST alone in all parameters. Conclusions: Well-designed CT regimens did not interfere with the development of physical fitness of trained team sports players.

## 1. Introduction

The most common team sports, such as soccer, rugby, American football, basketball, and handball are based on strength and endurance. Players need to develop multiple aspects of physical fitness to achieve the desired athletic performance in these sports. This necessitates players developing a well-rounded fitness to meet the physical demands of match-play. Within these criteria of physical fitness, maximal lower-body strength, power, and aerobic capacity are the most important determinants of athletic performance [1]. In a 90-min soccer match, for instance, the total distance a player runs ranges from 10–12 km at an average intensity of up to the anaerobic threshold (80–90% of maximum heart rate) [2], Within this context, they may be engaged in approximately 150–250 actions of 15–20 m of high-intensity sprints and perform numerous explosive actions [3,4]. These actions strongly influence the performance of players and the team, and can potentially change a match’s outcome [5]. Thus, in addition to constantly improving techniques and tactics, the development of physical fitness is currently being given greater consideration by strength and conditioning professionals. However, within current sporting contexts, the available training time may be extremely limited owing to congested competitive schedules [6]. It is difficult to schedule strength training (ST) and endurance training (ET) on alternate days. Strength and conditioning professionals must improve the time-efficiency of training on the premise and of guaranteeing the quality of training. Consequently, most team sports currently combined ST and ET on the same day to develop players’ physical fitness within routine training. Such a time-efficient training regimen that simultaneously combines ST and ET within a training cycle is called concurrent training [6,7,8,9,10,11,12,13]. Moreover, repeated-sprint training (RST) and sprint interval training (SIT) based high-intensity interval training (HIIT) methods are increasingly applied to team sports ET protocol, in view of the HIIT is considered one of the most effective and time efficient means for improving cardiorespiratory and metabolic function.

Since Robert C. Hickson first conducted concurrent training studies in 1980, an extensive body of research has been produced. Some studies have shown that concurrent training can potentiate the individual effects produced by ST and ET more than ST or ET alone [9,14,15,16,17]. For example, it was documented well that ST contributes to enhancing endurance performance by improving leg stiffness [18,19,20,21], which is a notion introduced from physics to characterize properties of certain types of deformable bodies under an influence of external forces [22,23,24,25,26]. However, other studies suggested that concurrent training compromises specific adaptive responses compared to ST or ET alone, more specifically, concurrent training may attenuate gains in muscle hypertrophy [27,28], strength [29,30,31], and power [32,33,34,35], but has little to no effect on endurance outcomes, such as VO_2_max or VO_2_peak and Yo-Yo test performance [11,12,35,36]. This phenomenon was defined as the “interference effect” of concurrent training [11,13,37,38]. However, to date, evidence for an interference effect has remained equivocal in humans. Previous studies have shown that variables such as intensity, volume, frequency, training status, ET protocol, organization order, duration of the recovery period, and nutrition supplements strongly influence individual adaptation to concurrent training. Although some meta-analyses [11,39,40,41,42,43,44] address the effects of the above-mentioned factors in athletic performance outcomes and physiological changes, there is a lack of robust evidence-based guidelines for trained team sports players.

Given the necessity for trained team sports players to simultaneously develop strength and endurance, as well as the popularity of HIIT-based endurance training in team sports. There is a need for a systematic review and meta-analysis to draw conclusions from the inconsistencies. The purpose of this study was to determine whether concurrent strength and HIIT-based endurance training (CT) would produce an interference effect on physical fitness development compared to ST or HIIT-based endurance training (HET) alone in trained team sports players. The hypothesis was as follows: ST carried out first in CT with an adequate interval time between ST and HET would not induce an interference effect on physical fitness development. Our findings will enhance understanding regarding the application of CT in team sports and assist strength and conditioning professionals to design better CT regimens.

## 2. Materials and Methods

This systematic review and meta-analysis was performed in line with the recommendations of the Preferred Reporting Items for Systematic Reviews and Meta-Analysis statement (PRISMA).

### 2.1. Literature Search Strategy

A search from 1980, the year that seminal research relating to concurrent training was published, to and including June 2022 was carried out using the following electronic databases: Web of Science, PubMed, and ScienceDirect. The search strategy used the following Boolean search syntax: “(concurrent training or concurrent exercise or combined training or concurrent strength and endurance training) and (soccer or football or association football or American football or rugby or basketball or handball or hockey or softball or team sports).” The search strategy is presented in Table 1. The search was limited to peer-reviewed English language articles. Following this, a primary exclusion based on titles and abstracts of retrieved articles was conducted individually by two authors (J.K. and Z.Y.) to assess their eligibility for review and meta-analysis. A secondary exclusion thereafter was also conducted individually by two authors (J.K. and X.Y.), based on a review of full-text articles. Any disagreements were solved by consensus with a third author (B.G.).

### 2.2. Eligibility Criteria

According to the Cochrane Collaboration methods, the PICOS (Participants, Intervention, Comparison, Outcomes, Study design) approach was used to build inclusion criteria for this systematic review and meta-analysis: (1) cohorts of team sports players aged >13 with no restriction for gender; (2) studies needed to have incorporated a concurrent training regimen, containing at least one group that followed single-mode ST/ET; (3) means and standard deviations of one or more outcome measures had to be reported for all groups pre- and post-intervention; (4) outcome measures included one or more of the following, lower-body 1-repetition maximum(1RM) of half squat, countermovement jump (CMJ) or squat jump (SJ), VO_2_max/peak or Yo-Yo test. These indicators represented lower-body maximum muscle strength, maximum immediate muscle power, and aerobic capacity, respectively; (5) randomized controlled or matched trials with at least 5 weeks of follow-up duration; (6) the original study.

### 2.3. Data Extraction

Two authors (X.Y. and Z.Y.) independently conducted data extraction from the included studies using an electronic data sheet. Data extracted comprised participant characteristics (including mean age, gender, and training status), study characteristics (including trial design, participant number, group set, intervention duration, training frequency, and exercise protocol), and outcome measures. Discrepancies about study conditions were discussed until a consensus was reached with a third author (B.G.).

### 2.4. Statistical Analyses

Though included studies reported multiple outcome measures, only those that were relevant to the meta-analysis were extracted. The differences in means in each group were calculated for each study using a comparison of mean change from pre- to post-intervention. The standard deviation (SD) of the mean change was also calculated and used to generate forest plots with study-specific point estimates and respective 95% confidence intervals (CIs). SD of the difference of the means was computed using the following formulation according to Deeks and Higgins [45]:

(1) $${\mathrm{SD}}_{\mathrm{change}}=\sqrt{\frac{\left(N_{T}-1\right){\mathrm{SD}}_{T}^{2}+\left(N_{C}-1\right){\mathrm{SD}}_{C}^{2}}{N_{T}+{\;N}_{C}-2}}$$

where N_T_, N_C,_ and SD_T_, SD_C_ represent the sample size in the experiment group and control group as well as the standard deviation of their responses, respectively. According to Morris [46], the SD_T_ and SD_C_ here use the standard deviation of the pretest mean of each group.

For each study, if the same measures were applied to the same outcome, pooled mean difference (MD) was calculated in Review Manager software (v.5.4. The Cochrane Collaboration, 2020). If not, between-group standardized mean differences (SMD) were calculated in Review Manager software using Hedges’ adjusted g, which is corrected for sample size. Analysis of the pooled data was conducted with a random-effects model, where weighting was based on inverse variance. According to Cohen, the overall effect size was interpreted as trivial (value < 0.2), small (0.2 ≤ value < 0.5), moderate (0.5 ≤ value < 0.8), or large (value ≥ 0.8), respectively. Statistical significance was considered for *p* ≤ 0.05. SMDs were also used to create funnel plots so that all estimates could be placed into one funnel plot. 

### 2.5. Quality Assessment

The Physiotherapy Evidence Database (PEDro) scale was used to rate the risk of bias and methodological quality of trials of the included studies. This rating scheme is reported as valid and reliable [47]. It quantified internal study validity using scoring from 0 (high risk of bias) to 10 (low risk of bias). Owing to the nature of sports training, it is often difficult to establish blinding within exercise interventions for both subjects and testing personnel. Thus, we modified the original version of the PEDro scale so that blinding of participants and investigators was not considered for quality assessment. The modified PEDro scale included an item indicating that the training load was controlled and reported, similar to the study of Ludyga et al. [48]. The scores of the seven included publications included studies ranged from 6 to 9, with an average score of 7.7, indicating moderate to high methodological quality (Table 2). 

**Figure 1:**
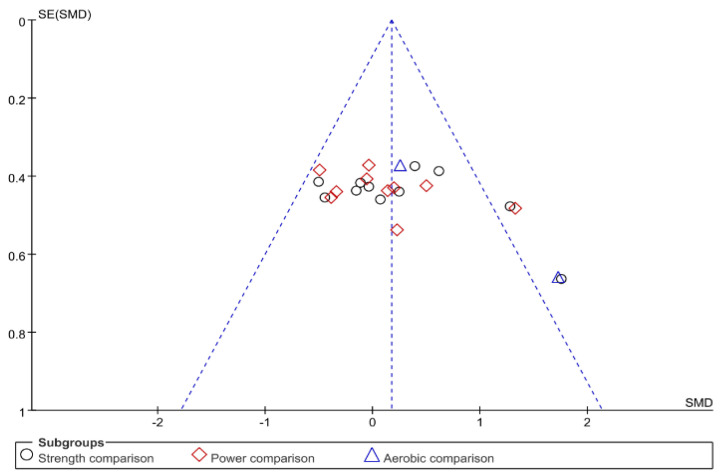
Funnel plots of standardized mean differences effect size versus standard error.

Heterogeneity between studies was assessed using I^2^ statistics for each outcome and interpreted as low, moderate, and high, corresponding to an I^2^ statistic of 25%, 50%, and 75%, respectively, according to Higgins et al. [49]. Furthermore, publication bias was assessed using funnel plots produced by Review Manager software. The funnel plot showed that the effects were relatively symmetrically distributed around the overall pooled effect size (Figure 1). Sensitivity analyses were also conducted to identify whether a particular study accounted for the heterogeneity.

### 2.6. Study Characteristics

**Figure 2:**
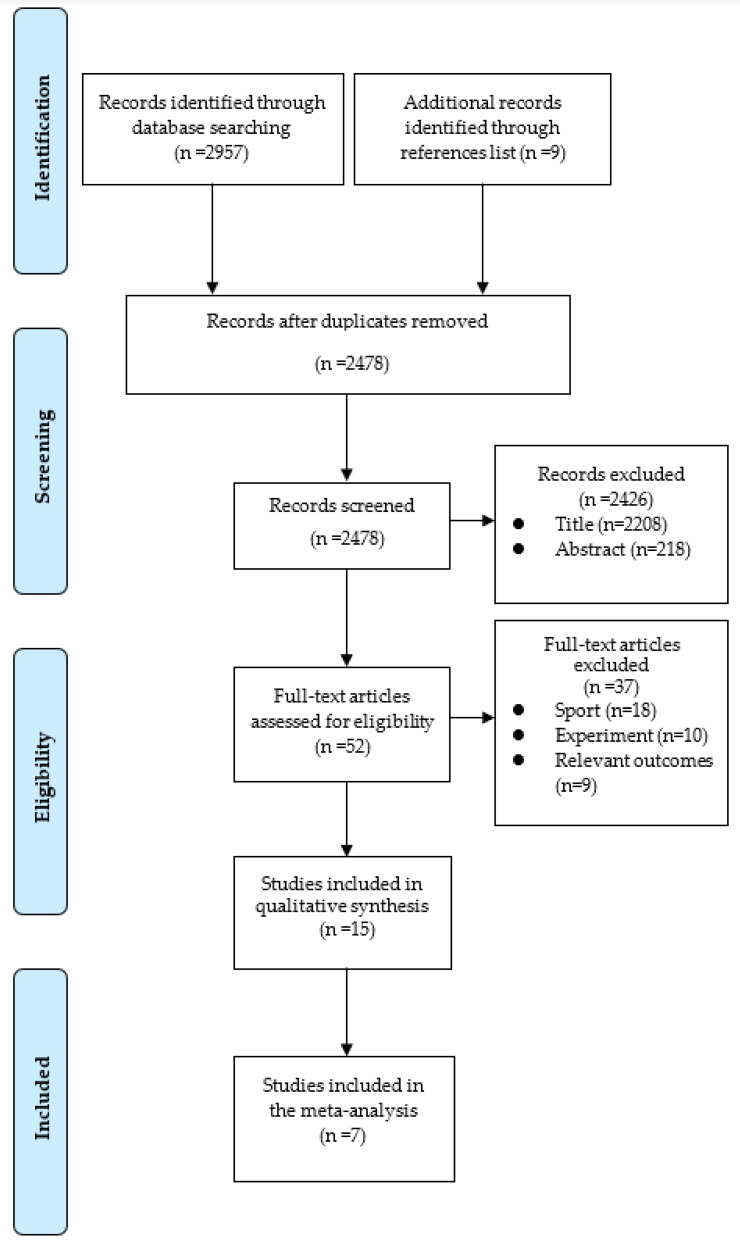
Flowchart of literature screening process.

Through systematic database searching, 1365, 921, and 671 relevant articles were initially identified in PubMed, Web of Science, and ScienceDirect, respectively. After duplicates were identified via Endnote software (v.9.3.3. Clarivate Analytics) and screening was performed based on the title and abstract, 52 articles remained. Through a manual search of the reference lists an additional nine studies were selected. After the initial full-text examination, 15 potential studies were further assessed. Further trial details of 15 studies were examined according to the eligibility criteria, and seven studies [7,9,50,51,52,53,54] were finally included in the systematic review and meta-analysis. Figure 2 shows the article selection process. All subjects in the seven studies were from six team sports: soccer, rugby, American football, basketball, and handball. Their training status was defined as “trained” and “athlete”, in accordance with Wilson et al. [11]. Five additional records [8,10,17,33,55] were not eligible for inclusion in the meta-analysis but were included in the qualitative analysis. In one study [51] of unreported data, the means and SDs of outcomes were estimated from figures using GetData Graph Digitizer (http://www.getdata-graph-digitizer.com/, accessed on 16 July 2022). In most studies the ET protocol was mainly repeated-sprint training (RST) and/or sprint interval training (SIT) HIIT models; the ST protocol included combined free weights, machine resistance, circuit resistance training, and plyometrics to maintain and develop maximum strength levels while further improving explosive strength. The intervention duration ranged from 5 to 12 weeks, and two to three times per week. More details can be found in Table 3.

## 3. Results

This systematic review and meta-analysis aimed to compare the effect of CT versus single-mode ST/HET on lower-body strength, power, and aerobic capacity development in trained team sports players, as well as the influence of internal organization order of CT on training adaptations. Upper-body performance was not included in the primary outcome analysis, because the ET protocols within CT in this study were for the lower extremities.

### 3.1. Lower-Body Strength

**Figure 3:**
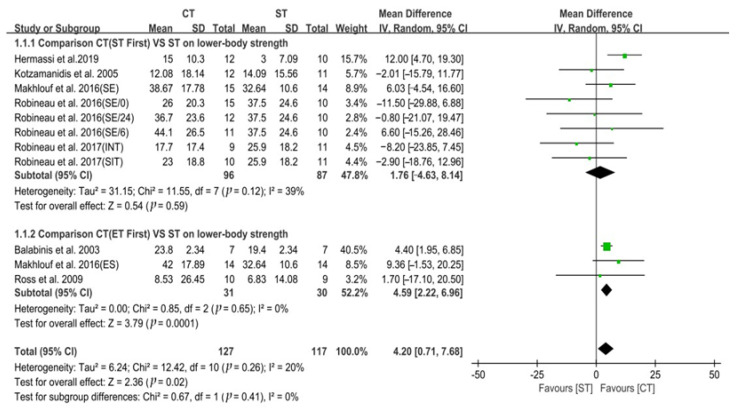
Forest plot comparing the effect of CT versus ST alone on lower-body strength [7,9,50,51,52,53,54].

Seven studies used 1 RM half squat as the outcome measure of lower-body strength. The results from 244 participants in the seven studies demonstrated that, compared to ST alone, CT had a significant positive effect on lower-body strength development in trained team sports players (*p* = 0.02). The heterogeneity was low (Chi^2^ = 12.42, *p* = 0.26; I^2^ = 20%). In the random effects model, aggregated MD and 95% CI were 4.20 kg (0.71, 7.68). Furthermore, a sub-group analysis on the internal organization order of CT revealed there was no statistically significant subgroup effect (Chi^2^ = 0.67, *p* = 0.41; I^2^ = 0%), suggesting that the internal organization order did not modify the effect of CT on the development of lower-body strength. However, a smaller number of trials and participants contributed data to the ET_first_ sub-group than to the ST_first_ sub-group. The data are shown in Figure 3.

### 3.2. Lower-Body Power

**Figure 4:**
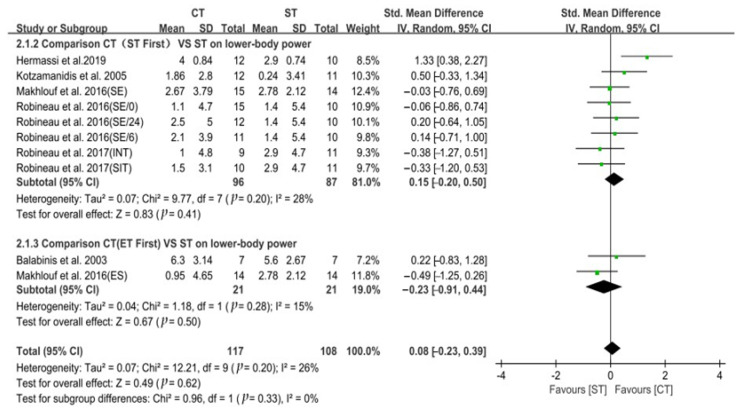
Forest plot comparing the effect of CT versus ST alone on lower-body power [7,9,50,52,53,54].

Six studies used CMJ or SJ as outcome measures of lower-body power. The six studies consisted of 225 participants and results suggested that there was no significant difference between CT and ST alone in the training adaptation of lower-body power of trained team sports players (*p* = 0.62). The heterogeneity was low (Chi^2^ = 12.21, *p* = 0.20; I^2^ = 26%). In the random effects model, aggregated effect size and 95% CI were 0.08 (−0.23, 0.39). A sub-analysis of the internal organization order revealed that there was no statistically significant subgroup effect (Chi^2^ = 0.96, *p* = 0.33; I^2^ = 0%), indicating that the internal organization order did not modify the effect of CT on power development. However, a smaller number of trials and participants contributed data to the ET_first_ sub-group than to the ST_first_ sub-group. More research should be carried out to establish more robust conclusions. The data are shown in Figure 4.

### 3.3. Aerobic Capacity

As only two of the included studies met the requirements for meta-analysis on aerobic capacity, for the sake of the robustness of the results, we did not perform a meta-analysis of aerobic capacity. However, based on the available literature, we made some relevant discussions below.

## 4. Discussion

The purpose of this study was to determine whether concurrent strength and HIIT-based endurance training would produce an interference effect on physical fitness development compared to ST or HET alone in trained team sports players. The primary findings of this meta-analysis indicate that there was no significant difference in developing lower-body power between CT and ST alone, but a significant difference was observed when compared with the effect of CT versus ST alone on lower-body strength. The internal organization order of CT did not significantly affect lower-body strength and power development. These findings were not in agreement with our hypothesis.

### 4.1. Lower-Body Strength

Regarding the development of lower-body strength, the present results demonstrated that CT had a small positive significant effect compared to ST alone. This was contrary to the concept of the universal nature of the so-called interference effect, in which Hickson first proposed that concurrent training reduces the ability to increase leg strength compared to ST alone [56]. Some researchers argue that Hickson’s study, with its high frequency and high load of training, induced an overtraining effect on participants, leading to a reduced change in skeletal muscle. They, therefore, conducted trials with lower training frequency and load, which suggested that peak torque in slow-velocity high-force regions produced significant improvements in both concurrent training and ST alone [27,34]. A highly cited meta-analysis also reported that concurrent training does not attenuate lower-body strength development compared to ST alone [11]. It is widely known that the potential for, and degree of, interference is related to concurrent training regimen, which involves the specific manipulation of various training- and non-training-related variables, such as training status of subjects, muscle phenotype of subjects, internal organization order of concurrent training, ET protocol, recovery length between ST and ET, nutrient supplements within the training session, and training period [12,13,55,57,58,59]. Therefore, any interference effects relative to concurrent training were complex. It cannot be considered to be only induced by the acute effects of a few factors and/or attributed simply to a molecular interference mechanism. It was originally thought that molecular signaling responses were incompatible in concurrent training regimens [60]. Namely, the activation of 5′-adenosine monophosphate-activated protein kinase (AMPK) induced by ET, which promotes mitochondrial protein synthesis–biogenesis and angiogenesis, will inhibit the mechanistic target of rapamycin complex 1 (mTORC1) by ST, which stimulates myofibrillar protein synthesis.

A recent study [61] that focused on the training status of subjects demonstrated that the addition of ET to an ST protocol may have a negative effect on lower-body strength development in trained, but not in moderately trained or untrained individuals. The study also points out that the impairment appears to be more pronounced when training is performed within the same session than in different sessions. Highlighting the specificity of training status, it seems reasonable to believe when previously sedentary or moderately active individuals participate in a concurrent training program, the response to the two exercise modes is additive and those exercise stimuli will initially induce a general adaptation. However, for trained athletes who already possess a high level of strength, there is less room for improvement and their volume of routine training is very large in contrast to sedentary or moderately active individuals. In addition, the rest interval between exercise bouts has been considered an important concurrent training-related variable [11]. Some investigators recently found that to maximize training adaptations there should be an adequate interval length between ST and ET within concurrent training; more specifically, if the aim is to maximize strength adaptations, the ST and ET bouts need to be separated by 3–6 h [11,42,43,53,55,62,63]. Most studies included in this meta-analysis separated ST and HET bouts by diverse interval times (these ranged from 10 min to 24 h) which may have greatly reduced the degree of residual fatigue caused by the prior exercise bout within concurrent training. In this instance, the internal organization order of concurrent training may not be an important consideration. Lee et al. [35] indicated that a 9-week concurrent training regimen with 3 h intervals between ST and ET, regardless of internal organization order, did not limit the development of lower-body strength compared to ST alone in healthy moderately-active men. In addition, the study of Ribeiro et al. [64] also supported our result that there was no significant difference regarding internal organization order of concurrent training with adequate interval times on strength development in soccer players. These findings highlight the importance of interval time, which provides a recovery window to manage prior exercise-induced residual fatigue, which is considered to subsequently limit the force-generating capacity of the muscle. Moreover, when observing the molecular mechanism, recent data suggest that combined high-intensity endurance exercise with ST not only does not inhibit mTORC1 signaling response in human muscles, but was also particularly effective for increasing mTORC1, compared with traditional continual endurance exercise [30,65].

Apró et al. [65] identified that phosphorylation of ribosomal protein S6 kinase 1 (S6K1), one of the best-characterized targets of mTORC1, was elevated ~five-fold immediately after high-intensity endurance exercise compared to baseline. Furthermore, phosphorylation of S6K1 in both concurrent training and single-mode ST was similarly and continuously increased from immediately after ST to 90-min and 180-min time points compared with baseline. In addition, the phosphorylation of eukaryotic elongation factor 2 (eEF2), a key component in protein translation machinery, reduced similarly after strength exercise under both conditions, which provides further support for the lack of AMPK inhibition on mTORC1 in human muscles. Helgerud et al. [66] indicated that 8 weeks of CT together with regular football training resulted in considerable improvement in elite-level professional players’ performance of the 1 RM half squat. It is worth noting that most CT groups of this meta-analysis implemented ET with HIIT models, such as RST and/or SIT. According to recent studies, RST and SIT models impose greater neuromuscular demands and higher glycolytic demands than other ET protocols [67,68,69]. Similarly, moderate-high intensity ST requires high muscle activation, force production, and glycolytic demand [70,71]. Apparently, there is a consistency factor associated with an increase in muscle strength between RST/SIT and ST. Furthermore, the team sports training frequency ratio of HET and ST in studies included in the present meta-analysis was between 2:1 and 3:1. One study suggests that a training frequency ratio between 2:1 and 3:1 leads to higher strength training-induced adaptations, compared with a ratio of 1:1 [72]. Consequently, the present study results show that HIIT-based concurrent training regimens not only do not produce antagonistic adaptations, but also have a significant positive effect on lower-body strength development compared to ST alone.

Taking the above together, the strength and conditioning professionals for team sports should be aware that good manipulation of various training-related variables, such as interval time, ET protocol, and training frequency ratio between ET and ST are important factors for developing players’ lower-body strength. However, the effect was pooled from only a few studies, so more research is needed to confirm the result.

### 4.2. Lower-Body Power

Unexpected results emerged when comparing the results of the studies that addressed power. While most studies suggested that concurrent training led to interference in power development compared to ST alone, such a claim was not supported in our meta-analysis which showed that there was no significant difference between CT and ST in the training adaptation in lower-body power of trained team sports players. Previous studies indicate that when concurrent training is performed with a high frequency of training (six to ten training sessions per week) it interferes with some indicators of explosive strength development, such as vertical jump [62] and angle-specific maximum torque at fast velocities of contraction [26]; the low-frequency concurrent training also leads to interference in explosive strength development [32]. It seems that power development may be more susceptible to the so-called interference effect of concurrent training than strength. As strength development was unaffected in this study and some others when comparing concurrent training to ST alone, we speculated that other neuromuscular mechanisms may account for it, just as previous researchers suggested probably mediated by a reduced improvement in rapid voluntary neural activation of the trained muscles [32]. In some previous studies, ST protocol was composed of heavy resistance, mainly aimed at developing lower-body maximum strength rather than explosive strength, for untrained subjects. However, training-induced adaptations are known to differ according to the specific mode of exercise used for ST. Therefore, this interference effect may hold true with regard to power development. Of note, the ST protocol for a trained population and athlete participants with superior strength levels in the studies included in our meta-analysis not only consisted of maximum strength drills, but also some plyometrics. Plyometrics are considered to be effective training exercises for developing explosive strength. In addition, almost all of these studies have adopted RST or SIT in ET programs. A recent study result showed that no significant difference in peak power was observed for ST alone and a concurrent training group in which ET consisted of SIT [73]. Therefore, we can rationally presume that in the studies included in our meta-analysis, ST protocols that use various forms of exercise to maintain and develop maximum strength and improve explosive power may not produce more neuromuscular fatigue than others. Furthermore, though the HET used in the studies included in our meta-analysis can induce greater neuromuscular demand and higher glycolytic demand, it is of short duration. Thus, combination exercises, such as ST protocols with HET with sufficient intervals may not impair power parameters, regardless of the internal organization order, as the current results reveal. Nevertheless, further research is needed to support these findings.

### 4.3. Aerobic Capacity

In terms of aerobic capacity, it was not able to perform a meta-analysis due to small sample sizes. However, the development of aerobic capacity may not be as susceptible to the so-called interference effect when HET combined with ST forms a concurrent training protocol according to available literature. Much evidence indicates that HIIT can be more effective for enhancing aerobic capacity compared with traditional continual endurance exercises of low-moderate intensity, long duration, and long-distance [74,75,76,77,78]. A previous study that involved eight weeks of CT together with regular soccer training resulted in an average 8.6% improvement in VO_2max_ of elite-level professional players [66]. Another study regarding CT on semi- and fully-professional soccer players identified a 19.4% improvement in the Yo-Yo intermittent recovery test (Level 2); furthermore, there was no significant difference in performance markers when altering the order of strength and endurance training in CT [17]. Based on previous studies it could explain that except for the role of HIIT itself, running economy, is a key parameter of the endurance performance of well-trained athletes. Through ST promoting triceps surae force and lower extremity muscle-tendon unit stiffness, that can improve stretch-shorten cycle efficiency, it is essential to the improvement of running economy; the most economical runners possess a higher contractile strength and greater tendon-aponeurosis stiffness in the triceps surae [79]. Thus, increased muscle-tendon unit stiffness and lower extremity contractile strength gained by ST seems to be an important factor contributing to the improvement of running economy after concurrent HIIT-based endurance training with ST in most team sports.

## 5. Limitations

Although this novel study provides insight to better understand CT regimens of team sports, some limitations should be acknowledged. One of the main drawbacks of this study was the available data from the literature concerning the physiological effect of CT on physical performance in team sports players were limited. Therefore, we were unable to conduct analysis on a greater number of indicators to assist in understanding the topic. In addition, as concurrent training effects are multifactorial, training- and non-training-related variables were important considerations when interpreting the outcomes of concurrent training interventions [58]; it is possible that any negative impacts may be ameliorated by manipulating some relevant variables. Hence, our conclusions are limited because within the included studies it was difficult to control all related variables to produce identical methodologies; this may have contributed to the relatively high degree of inconsistency for some indicators between the included studies.

## 6. Future Directions

More high-powered studies are needed to shed light on this intricate subject in team sports. Future studies should be well controlled and distinguish the training-related variables and non-training-related variables, especially in “real-world” daily routines of trainers and athletes, to classify whether any potentiating or interfering effect is caused by methodological factors or by a true underlying mechanism that we do not yet fully understand. Finally, studies of female athletes, especially high-level female players in team sports, should be enhanced so that any effect of sex on concurrent training adaptations can be determined.

## 7. Conclusions

Despite the limitations of this systematic review and meta-analysis, these findings can be utilized by strength and conditioning professionals to better understand and design concurrent training regimens which can assist players to maximize training adaptations relative to the match-play needs of a team sport. Based on our results, it seems that the physical fitness gains of trained team sports players may be optimized by the use of HIIT-based ET protocols in place of traditional ET protocols in team sport concurrent training regimens. Specifically, regardless of the internal organization order of CT, CT appears to have a significantly positive effect on the development of lower-body maximal strength in trained team sports players compared to ST alone, and does not interfere with their lower-body power development. To sum up, such concurrent training regimens can be time-efficient training protocols for developing physical fitness in trained team sports players.

## Figures and Tables

**Table 1:** Web of Science search strategy performed on 30 June 2022.

Concept Search Strategy	Line No.	Entry
Concurrent training	1	Concurrent training
	2	Concurrent exercise
	3	Combined training
	4	Concurrent strength and endurance training
	5	1 or 2 or 3 or 4
Sports	6	Soccer
	7	Football
	8	Association football
	9	American football
	10	Rugby
	11	Basketball
	12	Handball
	13	Hockey
	14	Softball
	15	Team sports
	16	6 or 7 or 8 or 9 or 10 or 11 or 12 or 13 or 14 or 15
	17	5 and 16

**Table 2:** Methodological quality assessment for inclusion in the study.

Study	1	2	3	4	5	6	7	8	9	10	Total
Balabinis et al., 2003 [9]	1	0	0	1	1	0	1	1	1	1	7
Kotzamanidis et al., 2005 [50]	1	1	1	1	1	0	1	1	1	1	9
Ross et al., 2009 [51]	1	1	0	0	1	0	1	0	1	1	6
Makhlouf et al., 2016 [52]	1	1	0	1	1	0	1	1	1	1	8
Robineau et al., 2016 [53]	1	1	0	1	1	0	1	1	1	1	8
Robineau et al., 2017 [7]	1	1	0	1	1	0	1	1	1	1	8
Hermassi et al., 2019 [54]	1	1	0	1	1	0	1	1	1	1	8

The modified PEDro scale item. 1. Eligibility criteria specified. 2. Random allocation. 3. Concealed allocation. 4. Groups similar at baseline. 5. Training load controlled and reported. 6. Assessor blinding. 7. Less than 15% dropouts. 8. Intention-to-treat analysis. 9. Between-group statistical comparisons. 10. Point measures and variability data. Each satisfied item contributes 1 point to the total PEDro score (range 0–10 points).

**Table 3:** Characteristics of the studies included in the meta-analysis.

Study	Sport	Training Status	Group	N(m/f)	Mean Age (Years)	ET			R E C	ST			Additional Training Except Intervention	Outcome Measure
						Protocol	W	F		Protocol	W	F		
Balabinis et al. 2003 [9]	Basketball	Trained	ET	7 (7/0)	22.4 ± 0.5	100 m, 200 m, 300 m, 400 m, 500 m sprints	7	4	—	—	—	—	Nothing	1 RM HS CMJ VO_2max_
			ET + ST	7 (7/0)	22.6 ± 0.8				7 h	Half squat, Bench press, Leg press, Lateral pull down	7	4		
			ST	7 (7/0)	22.2 ± 0.4	—	—	—	—					
			CON	5 (5/0)	22.2 ± 0.5	—	—	—	—	—	—	—		
Kotzamanidis et al. 2005 [50]	Soccer	Trained	ST + ET	12 (12/0)	17.0 ± 1.1	Maximal intensity repetitions of 30 m	9	2	10 min	Backwards lunge, Half squat, Hamstrings kick	9	2	Only the control group performed some moderate activity per week.	1 RM HS CMJ 30 m sprint
			ST	11 (11/0)	17.1 ± 1.1	—	—	—						
			CON	12 (12/0)	17.8 ± 0.3	—	—	—	—	—	—	—		
Ross et al. 2009 [51]	Soccer, American football	Athlete	ET	6 (6/0)	19.8 ± 1.8	8 to 12 maximal sprints for 40–60 m at 0–25% of each subject’s body mass	7	2	—	—	—	—	Subjects in all training groups refrained from participating in any type of exercise outside the domain of the study.	1 RM HS Power_peak_ 30 m sprint
			ET + ST	10 (10/0)	19.8 ± 1.2				N/A	Squat, Dead lift, Seated row, Dumbbell biceps curl, Leg extension, Core circuit, standing calf raise, Leg curl, Dumbbell hammer curl	7	2		
			ST	9 (9/0)	19.8 ± 1.4	—	—	—	—					
Makhlouf et al. 2016 [52]	Soccer	Athlete	ST + ET	15 (15/0)	13.7 ± 0.5	10 to 16 HIIT running without interruption according to the peak speed of each player	12	2	15 min	Bent over row, Push up, Forward lunge, Sit up, Upright row, Biceps curl, Supine leg raise, Front half squat, Stiff leg deadlift, Supine leg lateral twist, Weighted Forward lunge, Plyometrics	12	2	All players trained 4 times a week with a match. During the remaining weekly training sessions, players performed mainly technical-tactical drills.	1 RM HS CMJ YYIRT 10 m sprint 30 m sprint
			ET + ST	14 (14/0)	13.7 ± 0.5									
			AES	14 (14/0)	13.7 ± 0.5			4	AD			4		
			CON	14 (14/0)	13.7 ± 0.5	—	—	—	—	—	—	—		
Robineau et al. 2016 [53]	Rugby	Trained	ST + ET (0)	15 (N/A)	24.3 ± 3.8	Three 6-min sets at 120% individual maximal aerobic velocity of 15 s/15 s interval training	7	2	0 h	Bench row, Leg press, Half squat, Bench press, Plyometrics	7	2	Nothing	1 RM HS CMJ VO_2peak_
			ST + ET (6)	11 (N/A)	28.0 ± 4.5				6 h					
			ST + ET (24)	12 (N/A)	24.8 ± 3.9				24 h					
			ST	10 (N/A)	25.2 ± 4.4	—	—	—	—					
			CON	10 (N/A)	25.2 ± 3.5	—	—	—	—	—	—	—		
Robineau et al. 2017 [7]	Rugby	Trained	ST + ET (SIT)	10 (N/A)	26.4 ± 3.0	30 s runs at 100% individual maximal aerobic velocity with 30 s of active recovery, 30 s running all-out efforts with 4 min of passive recovery	8	2	24 h	Half squat, Bench row, Deadlift, Leg extension, Bench press	8	2	Nothing	1 RM HS CMJ VO_2peak_
			ST + ET (INT)	9 (N/A)	25.0 ± 3.7		—	—	—					
			ST	11 (N/A)	27.5 ± 2.5	—	—	—	—					
Hermassi et al. 2019 [54]	Handball	Trained	ST + ET	12 (12/0)	20.6 ± 0.5	Sub maximal 30 m shuttle runs, Small-sided games	10	2	0	Half squat, Drop jump, Pull over, Hurdle jumps, Medicine ball throw, Balance training, Bench press.	10	2	All participants performed their usual physical education training requirements.	1 RM HS CMJ

AD: Alternate day. AES: Alternate endurance and strength training. CON: Control group. CMJ: Countermovement jump. ET: Endurance training. F: Training frequency per week. HIIT: High intensity interval training. HS: Half squat. INT: Short interval training. INC: Incline. LEV: Level-grade. N(m/f): Number(male/female). N/A: Not available. REC: Recovery time between ST and ET. RST: Repeated sprint training. SIT: Sprint interval training. ST: Strength training. W: Weeks. YYIRT: Yo-Yo intermittent recovery test. 1 RM: One repetition maximum strength.

## Data Availability

GetData Graph Digitizer software is available at: http://getdata-graph-digitizer.com/download.php (accessed on 16 July 2022).
